# AlphaMissense prediction for the evaluation of missense variants in the diagnostic setting of neuromuscular disorders

**DOI:** 10.1177/22143602251370957

**Published:** 2025-09-05

**Authors:** Martin Krenn, Axel Schmidt, Matias Wagner, Margot Ernst, Elisabeth Graf, Gudrun Zulehner, Hakan Cetin, Fritz Zimprich, Jakob Rath

**Affiliations:** 1 Department of Neurology, 27271 Medical University of Vienna, Vienna, Austria; 2 Comprehensive Center for Clinical Neurosciences & Mental Health, 27271 Medical University of Vienna, Vienna, Austria; 3 Institute of Human Genetics, 9374 University of Bonn, Medical Faculty and University Hospital Bonn, Bonn, Germany; 4 Institute of Human Genetics, 9184 Klinikum rechts der Isar, School of Medicine, Technical University of Munich, Munich, Germany; 5 Institute of Neurogenomics, Helmholtz Zentrum München, Munich, Germany; 6 Department of Pathobiology of the Nervous System, Center for Brain Research, 31235 Medical University of Vienna, Vienna, Austria

**Keywords:** AlphaMissense, missense variants, neuromuscular disorder, next-generation sequencing

## Abstract

Next-generation sequencing has improved diagnostic outcomes for neuromuscular disorders, but interpreting rare missense variants remains challenging. We evaluated AlphaMissense, a recently developed machine learning tool, for predicting missense variant pathogenicity, using 45 (likely) pathogenic variants and 21 variants of uncertain significance from 58 deeply phenotyped patients. AlphaMissense predicted 69% of pathogenic variants correctly, but also classified 62% of variants of uncertain significance as pathogenic. Median AlphaMissense scores were not significantly different between pathogenic and uncertain variants. Overall, AlphaMissense accurately predicted the pathogenicity of most missense variants, but may be limited in certain functional contexts, highlighting the need for disease-specific interpretation approaches.

## Introduction

A substantial fraction of neuromuscular disorders (NMDs) is genetic in origin and attributable to monogenic variants. Despite the broad application of next-generation sequencing (NGS) in clinical practice, many patients remain undiagnosed because either no variants or variants of uncertain significance (VUS) are reported.^1–4^ These missense VUS pose a particular challenge given the abundance of human genetic variation, with only a small number of rare variants being clearly classifiable as pathogenic or benign.^5^

Computational (*in silico*) predictions provide a supportive level of evidence according to the variant interpretation guidelines of the American College of Medical Genetics and Genomics (ACMG).^6^ A quantitative framework has proposed new score thresholds for available *in silico* tools to achieve higher (i.e., moderate or strong) levels of evidence, aiming to reduce the number of missense VUS.^7^

AlphaMissense (AM), which combines unsupervised protein language modeling, structural protein information and population frequency data, has outperformed previous tools by providing pathogenicity scores for all possible amino acid substitutions across the entire human proteome. It classifies 89% of all possible missense variants as either pathogenic (32%) or benign (57%), achieving an accuracy of 90% when applied to a ClinVar dataset.^8,9^

In this study, we applied AM prediction to a set of diagnostically curated missense variants from adult NMD patients to evaluate its real-world utility within a diagnostic framework.

## Patients and methods

### Patients and next generation sequencing

We evaluated NMD patients who underwent diagnostic NGS at the Department of Neurology of the Medical University of Vienna, Austria, between 2015 and 2023. The ethics committee of the Medical University of Vienna approved the study (EC-Nr. 1201/2022 and 1021/2018). Diagnostic NGS was conducted at the Institute of Human Genetics of the Technical University of Munich, Germany. Details on sequencing, bioinformatics and data analysis have been reported elsewhere.^2^

### Variant classification and statistical analyses

Genetic variants were classified according to ACMG guidelines^6^ and modifications proposed by ClinGen (https://clinicalgenome.org/docs/, accessed on July 8, 2024), as previously reported.^2^ Strength of the ACMG PP3 criterion was determined by the REVEL score using previously suggested cut-off values.^7^ We evaluated all NMD-related (likely) pathogenic missense variants and reported missense VUS using AM prediction scores, ranging between 0 and 1. We used the thresholds suggested in the primary paper (<0.34: benign; >0.564: pathogenic; scores in between: ambiguous) for variant prediction.^8^ Two variants were excluded, because no AM score was available. In a further step, we compared the distribution of AM scores for (likely) pathogenic variants and VUS in our dataset with ClinVar-listed variants in disease genes from the gene panel ‘Other rare neuromuscular disorders’ (PanelApp, version 23.7, accessed in August 2024).

R (v4.3, 2023, R Foundation for Statistical Computing) and R Studio (v2023.6, 2023, RStudio PBC) were used for statistical analysis. Differences between continuous variables were compared using the Mann–Whitney U, Wilcoxon rank sum test or t-test, and differences between categorical variables using the chi-square or Fisher's exact test. Spearman correlation coefficients were calculated to analyze the relationship between AM and REVEL scores.

## Results

### Clinical and demographic characteristics

We evaluated 66 missense variants from 58 patients (28 females, 30 males; median age at NGS: 49 years, IQR 20.5). The leading phenotype was muscle disease (i.e., myopathies and myasthenic syndromes) in 30, hereditary spastic paraplegia in 10, neuropathy in 9, motor neuron disease in 6 and suspected mitochondrial disease in 3 patients. 39 patients (67%) had a disease onset in adulthood (≥18 years of age). Expected inheritance patterns of affected genes were autosomal recessive for 32 variants (48.5%), autosomal dominant for 31 variants (47%), and X-linked for 3 variants (4.5%).

### Genetic characterization of missense variants

**Figure 1.**
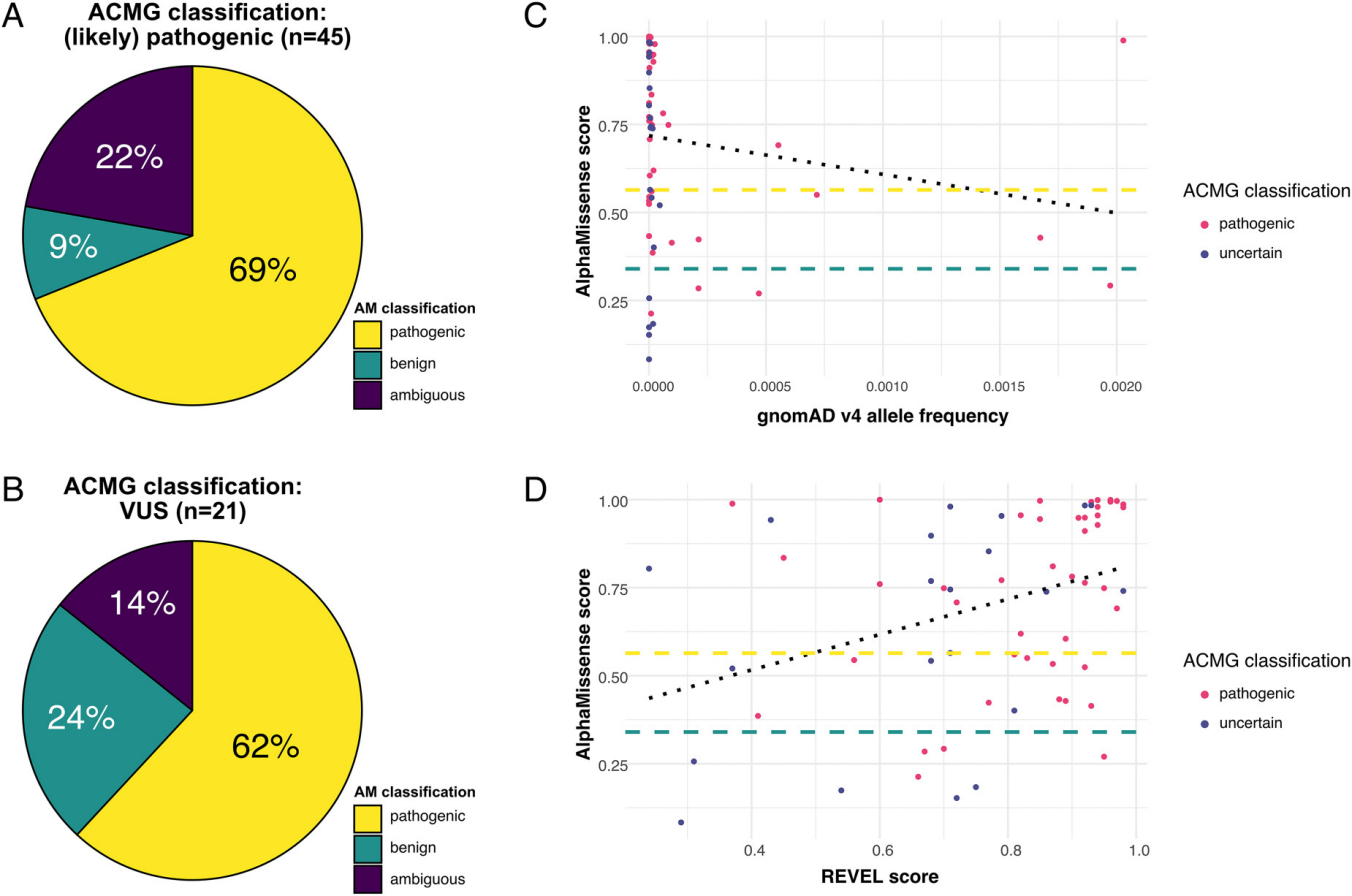
**Performance of AlphaMissense on curated variants from a deeply phenotyped adult neuromuscular cohort.** (A) Variant classification by AM for 45 (likely) pathogenic variants according to the ACMG criteria. (B) Variant classification by AM for 21 VUS according to ACMG criteria. (C) Comparison of AM scores with gnomAD allele frequency for each variant. (Likely) pathogenic variants are shown as black, VUS as yellow dots. The red dotted line represents the cutoff of the AM score for pathogenic variants (>0.564), the blue dotted line the cutoff for benign variants (<0.34). (D) Comparison of AM scores with REVEL scores. (Likely) pathogenic variants are shown as black, VUS as yellow dots. Note, that while both scores have continuous values between 0 and 1, values for each respective variant are not directly comparable because of different cutoffs for classification. The red dotted line represents the cutoff of the AM score for pathogenic variants (>0.564), the blue dotted line the cutoff for benign variants (<0.34). Abbreviations: ACMG, American College of Medical Genetics and Genomics; AlphaMissense, AM; gnomAD, Genome Aggregation Database; VUS, variants of uncertain significance.

The supplementary table provides a detailed overview of all reported missense variants including allele frequencies in gnomAD,^10^ number and classification of ClinVar entries,^9^ applied ACMG criteria, AM scores, AM prediction, REVEL scores and conservation metrics. 45 (68%) variants were classified as (likely) pathogenic and 21 (32%) as VUS according to the modified ACMG criteria. Of the 45 (likely) pathogenic variants, AM classified 69% (31/45) also as pathogenic, 22% (10/45) as ambiguous and 9% (4/45) as benign, respectively. Of the 21 VUS, AM classified 62% (13/21) as pathogenic, 14% (3/21) as ambiguous and 24% (5/21) as benign, respectively (Figure 1A and B). Stratified by mode of inheritance, AM classified 81% of autosomal dominant and 62% of autosomal recessive variants as pathogenic. This difference was not statistically significant (Fisher's exact test, p = 0.47). X-linked variants (n = 3) were excluded from this comparison due to the low number.

**Figure 2.**
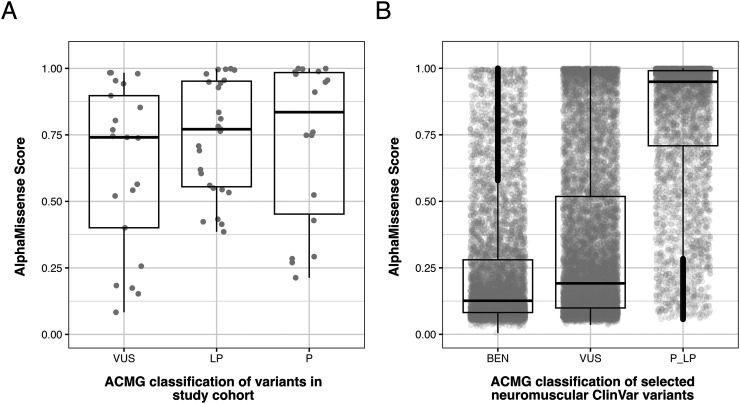
**Comparison of AlphaMissense scores between study cohort and ClinVar variants.** (A) Boxplots showing the distribution of AM scores for VUS, likely pathogenic and pathogenic variants in the study cohort. (B) Boxplots of AM scores for ClinVar variants categorized as benign, uncertain, and (likely) pathogenic from the ‘Other Rare Neuromuscular Disorders’ gene panel (PanelApp, version 23.7, data accessed in August 2024). Abbreviations: ACMG, American College of Medical Genetics and Genomics; BEN benign; LP, likely pathogenic; P, pathogenic; VUS, variants of uncertain significance.

The median AM score did not significantly differ between (likely) pathogenic (i.e., diagnostic) variants and VUS (0.77, IQR 0.43 and 0.74, IQR 0.50; p = 0.11). By contrast, AM scores of ClinVar-listed variants in NMD-relevant genes were remarkably lower for VUS than for (likely) pathogenic variants (Figure 2A + B). Compared to the ClinVar dataset, AM scores in our cohort were significantly higher for VUS but lower for (likely) pathogenic variants (p < 0.0001 and p = 0.025, respectively). In our dataset, all four (likely) pathogenic variants that were predicted as benign are well-established disease-causing variants (in the genes *SGCA*, *FKRP*, *BSCL2*) with multiple ClinVar entries as pathogenic and additional functional evidence.^11–14^ Furthermore, these four variants would have been classified as pathogenic without additional evidence from REVEL scores, which were used for the *in silico* ACMG criterion (PP3) in this study. The median allele frequency (AF) of falsely classified missense variants (gnomAD v4) was significantly higher compared to the other 41 pathogenic variants (0.0003406 vs. 0.0000031, p = 0.03, Figure 1C).^15^ A comprehensive *in silico* analysis revealed that the four false negative predictions may be explained by the amino acids’ roles either as glycosylation sites or as contributors to self-interaction in protein-packing or domain-domain contacts, where shape complementarity and specific interactions are crucial (see supplementary file for details). Finally, we observed a moderate positive correlation of AM scores with REVEL scores (r = 0.42; p = 0.0005, Figure 1D).

## Discussion

In this study, we evaluated the use of AM prediction on a set of diagnostically curated missense variants from a deeply phenotyped NMD cohort. We found that more than two-thirds of causative variants were correctly predicted as pathogenic by AM, while the remaining were misclassified as ambiguous or benign. The observed sensitivity was lower than expected, as suggested by the primary publication^8^ and real-life applications in hematological malignancies^16^ and cystic fibrosis.^17^ In contrast, a recently published paper on congenital myasthenic syndromes reported a lower accuracy of 64%.^18^

Somewhat surprisingly, the four disease-causing variants that AM incorrectly predicted as benign are well-established variants that fulfill ACMG criteria for pathogenicity regardless of *in silico* prediction. This discrepancy may at least partly be explained by the relatively high population frequencies of these variants, which are also taken into account by AM. Furthermore, computational predictions in general perform best on variants that cause a loss-of-function (LoF) of proteins.^19^ AM performance for non-LoF variants might still be superior compared to other computational predictors due to a better integrated use of structural information; however, it does not specifically predict the impact on protein-protein interactions or biophysical properties, which may negatively impact classification in dominantly inherited conditions. A recent work was dedicated to study AM performance in a range of different conditions,^20^ however, no clear trend has yet emerged to flag candidate false negative scores. Based on our observations, discrepant predictions between REVEL and AM warrant closer inspection.

Accurate application of computational predictors in a clinical context is crucial, as patients and clinicians often face remarkable uncertainty when dealing with VUS in diagnostic reports. In our study, the median AM score did not significantly differ between pathogenic variants and VUS. Given the restrictive reporting policy for VUS at our center, we hypothesize that many of these variants may indeed be causative, although they currently lack sufficient evidence for reclassification based on ACMG criteria. This finding is further supported by our comparative analysis using data from ClinVar (Figure 2A + B) and may reflect the utility of AM in prioritizing VUS for further study, rather than being interpreted as misclassification.

The main limitation of our study is the relatively small sample size, limiting the generalizability of our results. Nonetheless, our work may be particularly relevant for neuromuscular specialists, as we present a real-world dataset with highly curated variants obtained through a diagnostic approach. In addition, the recommended thresholds for AM classification are not aligned with the current ACMG/ClinGen framework for variant interpretation. Due to our limited cohort size, no conclusions can be drawn on specific score thresholds to determine pathogenicity in neuromuscular cohorts. Our findings only reflect the performance of the current version of AM, and future updates, including the integration of structural or functional data, may improve predictive accuracy. Further research may help validate AM scores more accurately in this context.

In conclusion, AM correctly predicts the pathogenicity of most known disease-causing missense variants in our real-world NMD cohort. However, its performance may be influenced by gene function and variant mechanisms, potentially leading to false negatives, e.g., depending on the affected amino acids’ roles either as glycosylation site or as contributors to self-interaction in protein packing or domain-domain contacts. We recommend using AM as part of a comprehensive variant assessment, particularly for prioritizing novel missense variants of uncertain significance. Further disease-specific validation studies may help establish optimal thresholds for genetic diagnostics.

## Supplemental Material

sj-xlsx-1-jnd-10.1177_22143602251370957 - Supplemental material for AlphaMissense prediction for the evaluation of missense variants in the diagnostic setting of neuromuscular disorders Supplemental material, sj-xlsx-1-jnd-10.1177_22143602251370957 for AlphaMissense prediction for the evaluation of missense variants in the diagnostic setting of neuromuscular disorders by Martin Krenn, Axel Schmidt, Matias Wagner, Margot Ernst, Elisabeth Graf, Gudrun Zulehner, Hakan Cetin, Fritz Zimprich and Jakob Rath in Journal of Neuromuscular Diseases

sj-docx-2-jnd-10.1177_22143602251370957 - Supplemental material for AlphaMissense prediction for the evaluation of missense variants in the diagnostic setting of neuromuscular disorders Supplemental material, sj-docx-2-jnd-10.1177_22143602251370957 for AlphaMissense prediction for the evaluation of missense variants in the diagnostic setting of neuromuscular disorders by Martin Krenn, Axel Schmidt, Matias Wagner, Margot Ernst, Elisabeth Graf, Gudrun Zulehner, Hakan Cetin, Fritz Zimprich and Jakob Rath in Journal of Neuromuscular Diseases

## Abbreviations

ACMG: American College of Medical Genetics and Genomics
AF: Allele frequency
AM: AlphaMissense
FAF: Filtering allele frequency
LoF: Loss-of-function
MND: Motor neuron disorders
NGS: Next-generation sequencing
NMD: Neuromuscular disorder(s)
VUS: Variant(s) of uncertain significance
WES: Whole-exome sequencing
WGS: Whole-genome sequencing
