# Supplementary material for: AlphaMissense prediction for the evaluation of missense variants in the diagnostic setting of neuromuscular disorders
Source: J Neuromuscul Dis. 2025 Sep 5:22143602251370957. Online ahead of print. doi: 10.1177/22143602251370957 (PMC13434959; doi:10.1177/22143602251370957)
Supplement: Supplementary file 1 [file sj-docx-2-jnd-10.1177_22143602251370957.docx]

Supplementary file

# CONTENT

[Supplementary methods	1](#_Toc198894532)

[Analysis of false negative predictions	1](#_Toc198894533)

[Seipin, encoded by BSCL2, Asn152Ser	1](#_Toc198894534)

[Ribitol 5-phosphate transferase, encoded by FKRP, Leu276Ile	3](#_Toc198894535)

[Alpha sarcoglycan, encoded by SGCA, Arg77Phe and Val247M	4](#_Toc198894536)

[Conclusion	6](#_Toc198894537)

[Supplementary References	7](#_Toc198894538)

# Supplementary methods

Sequence analysis was performed with the “align” tool provided at UniProt.^1^ Experimental structures, where available, were obtained from the protein data base (PDB) and depicted with Visual Molecular Dynamics (VMD).^2^ MetaRNN^3^ scores were obtained from the HOPE^4^ server.

# Analysis of false negative predictions

## Seipin, encoded by BSCL2, Asn152Ser

For this protein, splice variants exist and thus amino acid numbering is ambiguous. According to UniProt, isoform 4 has an Asn (N) in position 152.

In the canonical sequence, this amino acid is in position 88, see Figure below:

The difference in numbering is due to the longer N-terminal sequence of the “long” isoform. In the experimental structure^5^, the numbering corresponds to the “short” isoform, with the Asn in position 88. The only experimental structure at the PDB has the entry 6DS5. In this structure, Asn88 (corresponding to Asn152) is glycosylated – thus, for Asn152Ser, glycosylation can be expected to fail which may interfere with the assembly or function of the functional undecameric protein.

AM predicts for this amino acid exchange “likely benign” with a score of 0.213, while scoring all other possible substitutions in this position as likely pathogenic, see Figure below:

Legend: "y" denotes variants that are annotated or experimentally validated.

The MetaRNN score obtained from the HOPE server scores Asn152(88)Ser it with 0.683 rather towards the “likely pathogenic” end of the scale (0-1, higher values indicate likelihood for pathogenicity).

In summary, AM does not take glycosylation into account, which makes the predictions for glycosylation sites questionable.

## Ribitol 5-phosphate transferase, encoded by FKRP, Leu276Ile

The amino acid Leu276 is in a highly conserved position in the mammalian kingdom, see Figure below:

***Leu276 is highly conserved:*** *Sequence comparison of FKRP around Leu276 (red asterisk) using mouse, rat, macaca, chimp, bovine, dog, human and pig sequences from Uniprot.*

Several experimental structures are available.^6^

Leu276, located on the stem domain, contributes to the interface between the stem domain and the catalytic domain, see Figure below:

***Position of Leu276 at the domain interface:*** *Rendering of a single subunit from PDB entry 6L7T, the stem domain in orange and the catalytic domain in gold. Leu276 is highlighted in stick representation, amino acid sidechains in close contact shown as thin lines.*

As stated in Kuwabara et al. [6], *“replacement of Leu276 with Ile would affect the arrangement of the two domains”*. Thus, this amino acid contributes to an important self-interaction in the protein, in a role which is equivalent to protein-protein interactions where AM has been known to be less reliable.

## Alpha sarcoglycan, encoded by SGCA, Arg77Phe and Val247M

For this protein, no experimental structure is available. Close inspection of the AM predictions in the context of the AlphaFold predicted structure reveals that Arg77 (R77) contributes to the interface between two subdomains, and V247 contributes to hydrophobic packing. Both amino acids thus participate in self- interactions of the protein, see Figure below.

***Predicted structure of alpha sarcoglycan, positions of the variants: (A)*** *AlphaFold predicted structure with reliability scores provides an overview.* ***(B)*** *R77 and V247 are in the reliable portion of the model, which consists of two distinct subdomains. Amino acids 30-130 in gold, 131-255 in orange.* ***(C)*** *R77 is at the interface between the two subdomains.* ***(D)*** *V247 is well buried, and contributes to the packing of the beta- sheet to two alpha helices, where shape complementarity is crucial.*

For Arg77Cys (R77C), AM predicts “likely benign”, but interestingly, Arg77Ser (R77S) is predicted as “likely pathogenic”, see Figure below. The structural context suggests that the position Arg77 impacts strongly on the stability and/or mobility of the interface between the subdomains and thus variants may have higher impact than predicted.

Legend: "y" denotes variants that are annotated or experimentally validated.

For Val247M (V247M), AM prediction is “likely benign” with a score of 0.285, which brings it close to the “ambiguous” range. In a region of structural packing the larger size of methionine may interfere with shape complementarity, and thus structural considerations would result in a worse score – the metaRNN score from the HOPE server is 0.649 (0-1, larger numbers indicate higher likelihood for pathogenicity) is accordingly higher.

Legend: "y" denotes variants that are annotated or experimentally validated.

# Conclusion

In total, the four false negative predictions can be rationalized on the basis of the respective roles of the affected amino acids either as glycosylation site, or as contributors to self-interaction in either protein-packing or domain-domain contacts, where shape complementarity and specific interactions are very important.

# Supplementary References

1	UniProt, C. UniProt: the Universal Protein Knowledgebase in 2025. *Nucleic Acids Res* **53**, D609-D617 (2025). <https://doi.org/10.1093/nar/gkae1010>. Accessible through <https://www.uniprot.org/>

2	Humphrey, W., Dalke, A. & Schulten, K. VMD: visual molecular dynamics. *J Mol Graph* **14**, 33-38, 27-38 (1996). <https://doi.org/10.1016/0263-7855(96)00018-5>. Accessible through <https://www.ks.uiuc.edu/Research/vmd/>

3	Li, C., Zhi, D., Wang, K. & Liu, X. MetaRNN: differentiating rare pathogenic and rare benign missense SNVs and InDels using deep learning. *Genome Med* **14**, 115 (2022). <https://doi.org/10.1186/s13073-022-01120-z>

4	Venselaar, H., Te Beek, T. A., Kuipers, R. K., Hekkelman, M. L. & Vriend, G. Protein structure analysis of mutations causing inheritable diseases. An e-Science approach with life scientist friendly interfaces. *BMC Bioinformatics* **11**, 548 (2010). <https://doi.org/10.1186/1471-2105-11-548>. Accessible through <https://www3.cmbi.umcn.nl/hope/>

5	Yan, R. *et al.* Human SEIPIN Binds Anionic Phospholipids. *Dev Cell* **47**, 248-256 e244 (2018). <https://doi.org/10.1016/j.devcel.2018.09.010>

6	Kuwabara, N. *et al.* Crystal structures of fukutin-related protein (FKRP), a ribitol-phosphate transferase related to muscular dystrophy. *Nat Commun* **11**, 303 (2020). <https://doi.org/10.1038/s41467-019-14220-z>
